# Colour vision in thrips (Thysanoptera)

**DOI:** 10.1098/rstb.2021.0282

**Published:** 2022-10-24

**Authors:** Karla Lopez-Reyes, Karen F. Armstrong, Robert W. H. M. van Tol, David A. J. Teulon, Michael J. Bok

**Affiliations:** ^1^ Bio-Protection Research Centre, Lincoln University, Lincoln, New Zealand; ^2^ Better Border Biosecurity (B3, B3nz.org.nz), New Zealand; ^3^ Biointeractions and Plant Health (BIONT), Wageningen University and Research, Wageningen, The Netherlands; ^4^ BugResearch Consultancy, The Netherlands; ^5^ The New Zealand Institute for Plant and Food Research Limited, Auckland, New Zealand; ^6^ Lund Vision Group, Department of Biology, Lund University, Lund, Sweden

**Keywords:** visual ecology, pest control, eyes, photoreceptors, behaviour, colour response

## Abstract

Insects are an astonishingly successful and diverse group, occupying the gamut of habitats and lifestyle niches. They represent the vast majority of described species and total terrestrial animal biomass on the planet. Their success is in part owed to their sophisticated visual systems, including colour vision, which drive a variety of complex behaviours. However, the majority of research on insect vision has focused on only a few model organisms including flies, honeybees and butterflies. Especially understudied are phytophagous insects, such as diminutive thrips (Thysanoptera), in spite of their damage to agriculture. Thrips display robust yet variable colour-specific responses despite their miniaturized eyes, but little is known about the physiological and ecological basis of their visual systems. Here, we review the known visual behavioural information about thrips and the few physiological studies regarding their eyes. Eye structure, spectral sensitivity, opsin genes and the presence of putative colour filters in certain ommatidia strongly imply dynamic visual capabilities. Finally, we discuss the major gaps in knowledge that remain for a better understanding of the visual system of thrips and why bridging these gaps is important for expanding new possibilities for applied pest management strategies for these tiny insects.

This article is part of the theme issue ‘Understanding colour vision: molecular, physiological, neuronal and behavioural studies in arthropods’.

## 1. Introduction

Since the discovery of colour vision in bees a little over a century ago, many important advances have been made to expand our understanding of insect colour vision and visually guided behaviours [1]. However, insects are a remarkably diverse group, and much of the information on their colour vision has historically come from more commonly studied insect orders and a few model species, such as honeybees (Hymenoptera), *Drosophila* (Diptera) and some species of moths and butterflies (Lepidoptera), although information on other insects such as crickets (Orthoptera), dragonflies (Odonata), beetles (Coleoptera) and cockroaches (Blattodea) can also be found in the literature. Still, very little is known about colour vision in other insect groups, particularly herbivorous pests, despite a wide interest in using their behavioural response to attractive visual cues for their monitoring and control in agriculture [2–10]. One such example is thrips (Thysanoptera), a group of small fringed-winged insects (approx. 1–2 mm long) which include several species that are economically important, damaging pests for various crops [11–13].

**Figure 1.**
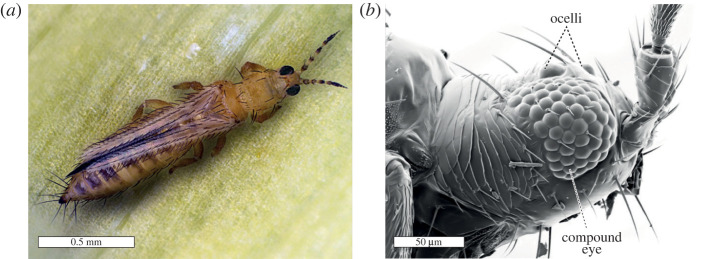
Eyes of the WFT (*Frankliniella occidentalis*). (*a*) Female *F. occidentalis* on a leaf. Photo by Dr. Manfred Ulitzka. (*b*) Scanning electron micrograph of a female *F. occidentalis* head indicating a compound eye and two of the three dorsal ocelli.

There are around 6000 identified species of thrips with highly diverse ecologies, including herbivores that feed on plant tissue and pollen (sometimes also acting as pollinators), fungivores, predators, parasites, and even eusocial and sub-social species, some of which are gall-forming [14,15]. Many Thysanoptera species, mainly belonging to the suborder Terebrantia, live on flowers and feed by piercing plant tissue and sucking cell contents [14,16–18]. Despite the diversity present in the group, to date the majority of studies on thrips have been limited to a few invasive, polyphagous pest species of terebrantids, which are known to transmit damaging tospoviruses when feeding from plants [13,19,20]. This review on thrips colour vision is therefore inevitably constrained to the literature of pest species. Among these, the best studied is the western flower thrips (WFT), *Frankliniella occidentalis* (Pergande), which has achieved worldwide distribution and causes severe economic impacts [21,22] (figure 1*a*).

Cumulatively, knowledge to date reveals that thrips appear to maintain complex colour discrimination capabilities despite extreme miniaturization of their compound eyes, raising tantalizing questions about their retinal physiology. However, conclusions from the many behavioural studies on the function of colour vision in this group are difficult to interpret due to their diverse methodologies and outcomes. This review aims to organize and consolidate the behavioural information about thrips visual attraction alongside physiological studies in order to better understand thrips colour vision in an ecological context. We discuss the major gaps in knowledge that remain and propose research directions that could lead to a better understanding of the thrips visual system and progress towards applied pest management strategies.

## 2. Thrips eyes

### (a) Anatomy and optics

Thrips have a pair of rounded, crescent-shaped compound eyes prominently positioned laterally on the head as well as three dorsomedial ocelli in winged species (figure 1*b*). In extremely small flying insects such as thrips, miniaturization of the anatomy exerts great pressures and constraints on the organization of the compound eyes while maintaining optical functionality for dynamic visual behaviours [23–25]. Extreme size constraints inevitably have an impact on eye architecture and resolution, as any adaptation will be limited by the available space. Furthermore, the corneal and crystalline cone lenses of each ommatidium must be large enough to overcome the optical thresholds required to properly focus light into the photoreceptor rhabdoms [26]. Consequently, despite the eyes occupying a significant volume of the head, thrips typically have only around 60–70 ommatidia in each eye, compared to a median of 165 in aphids (2–5 mm body length) [27,28], over 700 in *Drosophila* (approx. 3 mm body length) [29,30] and up to 10 000 in honeybees (workers approximately 15 mm body length) [31,32]. Some wingless species such as the soil thrips (*Bebelothrips*) possess fewer than 10 ommatidia [18,33], a reduction that is likely related to a de-emphasis in visual behaviours.

Little is known about the internal anatomy or ommatidial structure of the thrips eye. Mazokhin-Porshnyakov & Kazyakina [33] reported that the flower-dwelling *Thrips physapus* has apposition compound eyes. Below the cuticular cornea, each ommatidium is composed of a tetrapartite crystalline cone atop a fused rhabdom formed from seven retinular cells. The rhabdoms widen distally and the dorsal ommatidia are shorter than those in the ventral hemisphere. However, it is unknown if there are additional structural adaptations to the ommatidia allowing them to function as a dorsal rim for celestial polarization orientation, common in diurnal insects [34].

**Figure 2.**
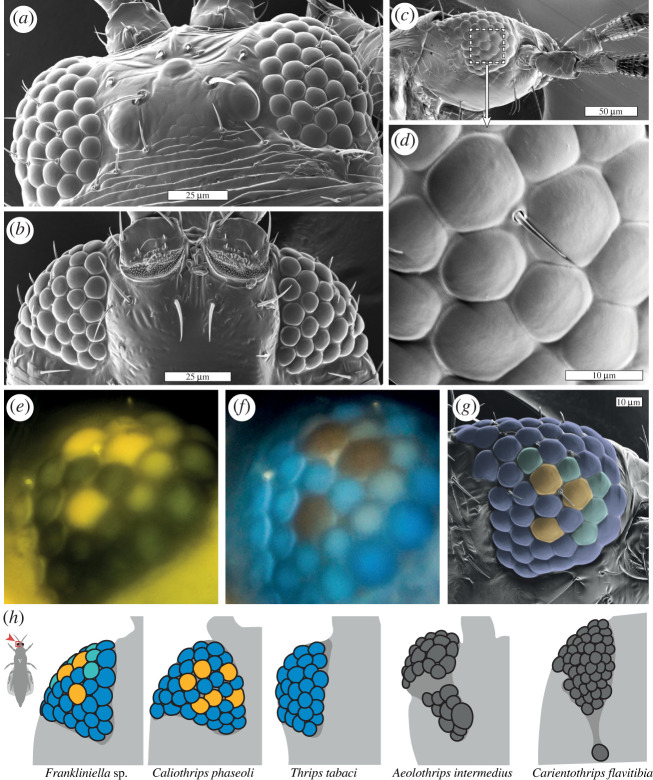
Putative visual specializations in the eyes of thrips. Dorsal (*a*) and ventral (*b*) SEMs of the head of *Frankliniella* sp. with the anterior of the thrips oriented upwards on the page. (*c*) A side-oriented SEM of the head of *Frankliniella* sp. with a dashed box indicating the region enlarged in (*d*). Auto-fluorescence micrographs of the ventral eye of *Frankliniella* sp. under blue (*e*) and UV (*f*) illumination. Fluorescence is indicative of light absorption by putative spectral filter pigments in subsets of the ommatidia. (*g*) A SEM of *Frankliniella* sp. with facets coloured to indicate the locations of blue-absorbing/yellow-emitting fluorophores (yellow), UV-absorbing/blue-emitting fluorophores (blue) and both fluorophores (teal). (*h*) Diagrams of the ventral eye from five thrips species with facets coloured for species where the presence of fluorophore pigments have been observed. *Frankliniella* sp. and *Thrips tabaci*: M Bok 2019, unpublished observation. *Caliothrips phaseoli* after Mazza *et al*. [35]. *Aeolothrips intermedius* redrawn from Moritz [36] and *Carientothrips flavitibia* redrawn from Eow *et al*. [37]. Details about the micrography in this figure can be found in the electronic supplementary material, Methods.

Thrips are expected to also possess poor resolving power based on the small number and size of ommatidia. In *Thrips physapus*, for instance, the interommatidial angle ranges between 11° and 15° [33]. In comparison, the maximum interommatidial angle is 8° in aphids [27], 5° in *Drosophila* and approximately 1° in honeybees [29]. Despite this, thrips show some intriguing regionalization of structural adaptations in the eyes. Though some thrips appear to possess uniformly sized facets across the entire eye, many species have variable numbers and arrangements of enlarged facets positioned on the ventral hemisphere of the eye, with more uniform facets on the dorsal region (figure 2*a–d*). In other species, such as the facultative predator *Aeolothrips intermedius*, the eyes elongate ventrally and project a separated cluster of ommatidia, including a single, greatly enlarged facet, under the head [36] (figure 2*h*). Ommatidial specialization in the ventral eye regions of flying insects suggests a role related to food search behaviours. In the glasshouse whitefly (*Trialeurodes vaporarorium*) for example, differences in ommatidial size and spectral sensitivity have been found between the dorsal and ventral regions of the eye, with the dorsal region having more, smaller ommatidia and increased ultraviolet (UV) sensitivity to aid in celestial orientation and navigation. By contrast, the ventral part of *T. vaporariorum* eyes present fewer, larger ommatidia that are tuned towards longer wavelengths in the green-yellow region of the light spectrum, presumably for host-searching [38].

### (b) Spectral filtering

Spectral filtering that contributes to colour vision is widespread in arthropods [39] and is perhaps most dramatically evidenced in mantis shrimp [40] and butterflies [41]. The incorporation of pigments or other materials, which selectively attenuate the wavelengths of light through the optical path of specific ommatidia, can shift, narrow or even multiply the spectral sensitivities of their associated photoreceptors. There is some evidence supporting the existence of spectral tuning mechanisms in thrips. Many species of thrips have a constellation of between two and seven enlarged facets in the ventral eye containing an optically dense, yellow-tinted pigment, likely sequestered in the cornea or crystalline cones [42–44]. In *Frankliniella* sp., this pigment absorbs blue light and emits yellow fluorescence, allowing it to be visualized with fluorescent microscopy in intact individuals (figure 2*e*). The remaining facets contain a fluorophore that absorbs UV light and emits blue light (figure 2*f*), with some apparent overlap of fluorescent pigments in a few facets (figure 2*g*). This UV-absorbing pigment has also been observed in *Caliothrips phaseoli* [35] and *Thrips tabaci* (onion thrips) (M Bok 2019, unpublished observation), though the latter apparently lacks enlarged ventral facets with the yellow pigment (figure 2*h*).

Mazza *et al*. [35] suggest that the UV-absorbing pigment acts as a short-pass filter for UV photoreceptors, absorbing UV light above 350 nm in wavelength and shifting photoreceptor sensitivity and behavioural response into the UV-B range (less than or equal to 315 nm). The use of short-pass UV filters to tune photoreceptor sensitivity to such short wavelengths has otherwise only been observed in mantis shrimp [45,46]. Unfortunately, it is difficult to infer the true optical significance of the blue- and UV-absorbing pigments in thrips eyes until their absorption spectra are measured and compared with the sensitivities of their associated photoreceptors.

### (c) Spectral sensitivity of thrips eyes

**Figure 3.**
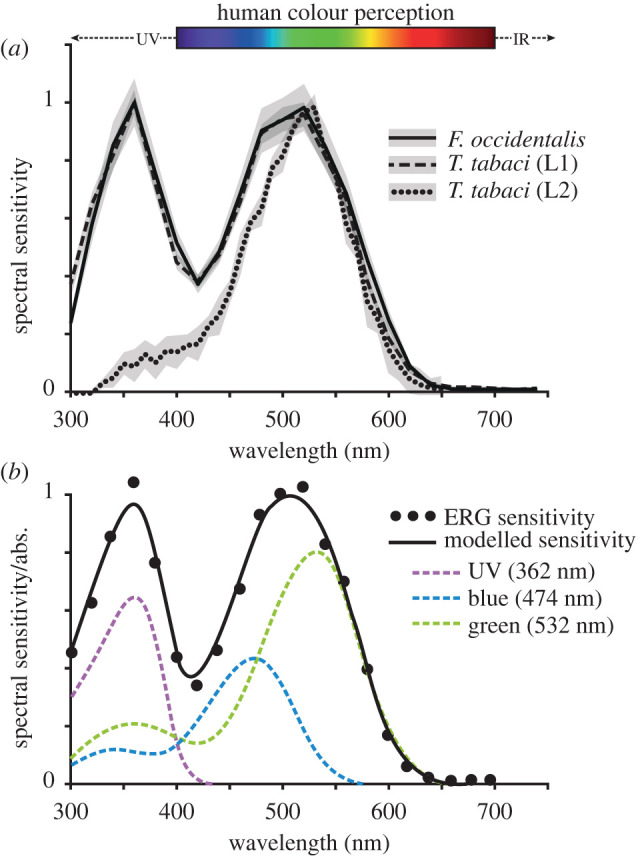
Spectral sensitivity of thrips. (*a*) Whole-eye ERG spectral sensitivity curves (normalized ± s.d.) of *F. occidentalis* (solid line), *T. tabaci* biotype L1 (dashed line) and *T. tabaci* biotype L2 (dotted line) adapted from Makabe *et al*. [49], Otani *et al*. [48] and Egri *et al*. [50]. (*b*) Modelled spectral sensitivity from *F. occidentalis* (black line) produced by summation of three visual pigment absorbance curves (coloured dashed lines) to fit the ERG spectral sensitivity results (circles). Adapted from Makabe *et al*. [49].

No intracellular recordings of individual thrips photoreceptors are available. However, a handful of electroretinogram (ERG) studies exist for three species, *F. occidentalis* [47,48], *T. tabaci* (L1 and L2 biotypes) [49,50] and *Scirtothrips dorsalis* [51]. From these ERG studies, sensitivity of the whole retina appears quite similar across all three Thripidae species, and between males and females. There are two distinctive peaks, one in the UV region with maximum response around 360–365 nm and another in the green region (figure 3*a*). Modelling of visual pigments template spectra to match these results suggests that the sensitivity curve is produced by three visual pigments peaking in the UV, blue and green [48,49] (figure 3*b*). This agrees with the presence of long-wavelength-sensitive (LWS) and short-wavelength-sensitive (SWS) opsin genes, one belonging to the UV clade (SWS-UV) and one to the blue clade (SWS-B), described below, and is consistent with many trichromatic insect colour vision systems [1]. Interestingly, the L2 biotype of *T. tabaci* has a unimodal sensitivity curve lacking the UV peak [50] (figure 3*a*). The green peak varies slightly between species and studies: 535 nm [47] or 500 nm [48] for *F. occidentalis* and the L1 biotype of *T. tabaci* [49], and 520 nm for *S. dorsalis* [51].

Trichromacy in thrips would be consistent with many insects, including the green peach aphid (*Myzus persicae*), the pea aphid (*Acyrthosiphon pisum*) [52,53] and the greenhouse whitefly (*Trialeurodes vaporarorium*) [54], all of which are also phytophagous insects that belong to the sister order Hemiptera [55]. For aphids and whitefly, trichromacy is explained by the putative blue receptor peak being close to that of the green receptor, and consequently, they appear as a single, broad peak in ERG measurements [52,54]. A similar scenario is likely for thrips.

### (d) Opsins genes

**Figure 4.**
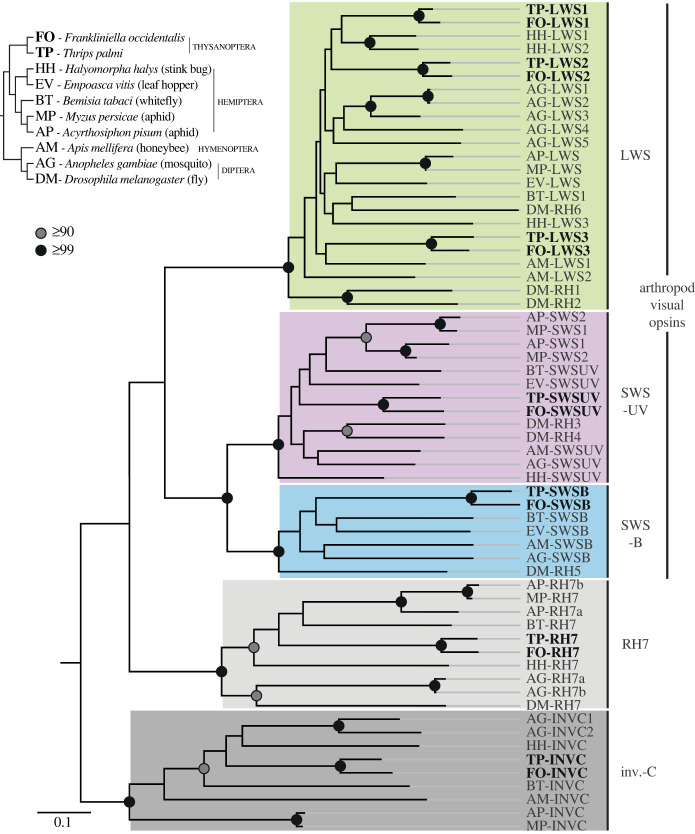
Opsin genes found in the genomes of *F. occidentalis* and *T. palmi*. Phylogenetic tree of insect opsin sequences. *F. occidentalis* (FO) and *T. palmi* (TP) sequences are bolded. Branch bootstrap supports are indicated as black circles (greater than or equal to 99%) or grey circles (greater than or equal to 90%). Supports below 90% are omitted. Other insect species included in the tree are indicated in the insect species cladogram at the top left. Opsin clades included in the phylogeny: visual opsins: LWS, SWS-B, SWS-UV; and non-visual opsins: rhodopsin 7 (RH7) and invertebrate ciliary opsins (inv.-C). Details on phylogenetic reconstruction can be found in the electronic supplementary material, Methods.

Genome sequencing in two species of thrips, *F. occidentalis* [56] and *Thrips palmi* [57]*,* has revealed some information regarding the opsins that may be expressed in the photoreceptors of their eyes. Both genomes contain at least seven opsin genes, five of which belong to clades typically used in insect visual systems (figure 4) [58]. The other two belong to the rhodopsin 7 (RH7) and invertebrate ciliary opsin (inv.-C, referred to as pteropsin in insects) clades whose functions remain obscure and are both thought to typically be expressed in brain photoreceptors [59,60]. Of the five visual opsins, there are three LWS opsin genes and two SWS opsin genes, SWS-UV and SWS-B. The SWS-UV opsin has a lysine residue at position 90 (relative to bovine rhodopsin), indicative of UV spectral tuning in arthropod visual opsins [61]. It is also possible that one of the LWS opsins is specialized for expression in the ocelli [62]. Further work is needed to determine if and where each of the opsins is expressed in the thrips eye.

### (e) Conclusion regarding eye structure and functions

Thrips eyes, despite their small size and limited resolving power, exhibit a number of adaptations that suggest these insects retain elaborate visual capabilities in a highly miniaturized package. There remains much to explore regarding these eyes, including the localization of opsin gene expression, photoreceptor fine structure and arrangement, absorptive properties of the putative spectral filters, and the electrophysiological responses of individual photoreceptors. Furthermore, the diversity in eye anatomy and ventral filter patterns in different species of thrips hints at fascinating visual specializations that may be related to host plant selection or other ecological and lifestyle factors. Thus, it is unsurprising that, as we will expand upon in the next section, thrips exhibit robust visually guided behaviours and complex colour discrimination capabilities.

## 3. Behavioural responses to colour in thrips

**Figure 5.**
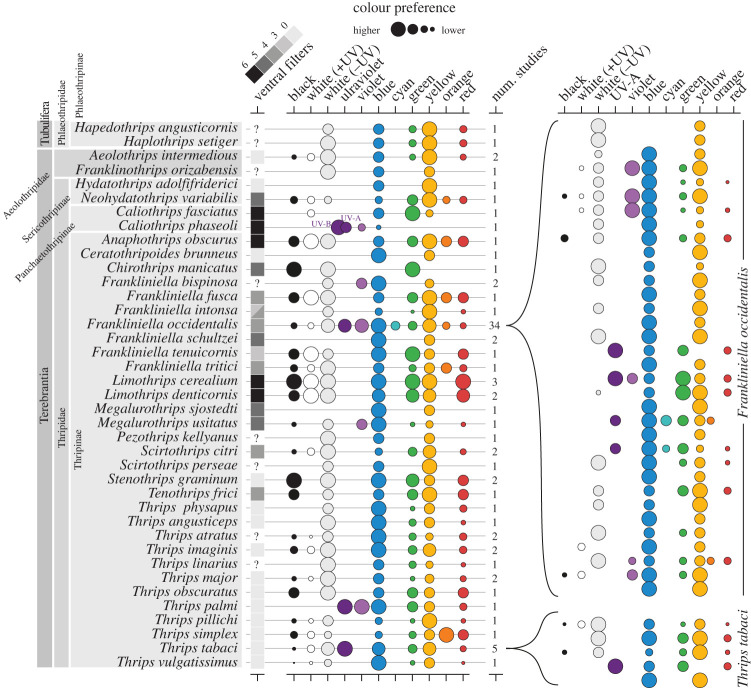
Colour attraction in thrips species generalized according to various behavioural experiments. For each study, we represent the relative preference of the tested colours with circles (larger circles indicate greater preference and are qualitatively derived from preference rankings described in the electronic supplementary material, table S1; see the table description in the electronic supplementary materials for additional details). For species where multiple studies have been conducted, the preferences were averaged. The two most-studied species, *Frankliniella occidentalis* and *Thrips tabaci*, are expanded on the right to highlight variation in testing conditions and preference results across multiple studies. The number of ventral ommatidia containing putative yellow (blue absorbing) spectral filters for each species is also indicated according to Nakahara [42].

Many thrips exhibit visually guided behaviours related to orientation towards host plants or habitats where they feed, mate, lay their eggs and sometimes even aid in pollination [17,63]. We assume that the behavioural response of thrips to visual stimuli, including colour, is through attraction (i.e. oriented movement towards the stimuli). One of the earlier studies evaluating colour attraction of thrips was published by Lewis [64], who incorporated colour into an assessment of different trap types for population monitoring. Since then, most colour vision information about thrips has continued to be similarly inferred from studies measuring attraction to coloured traps based on the number of thrips caught. A synthesis of the literature describing colour preferences confirms that many species of thrips are attracted by colour, but not all colours are preferred equally, and preference varies between species, populations and experimental conditions (figure 5; electronic supplementary material, table S1).

By our best count, colour attraction or preference experiments have been performed on at least 39 species of thrips, with many of these belonging to the Thripidae family due to interest in their plant pest status. Unfortunately, few species with non-herbivorous lifestyles have been considered, making ecological comparisons difficult. Most species have only been examined in one or two studies, except *T. tabaci* with five, and *F. occidentalis* with 34. These studies indicate that thrips are attracted to various colours. However, it is difficult to make broad generalizations about the colour preference of thrips, both within and between species, due to the wide variety of experimental conditions and their sometimes-contradictory results (electronic supplementary material, table S1). Furthermore, the experiments are often biased towards a focus on blue and yellow stimuli, which have been heavily favoured historically and commercially as the attractant in trap design. Experiments that accurately test a wide spectrum of colour cues including UV are limited, though more recent efforts have improved in this regard due to the wide availability of spectrally flexible and narrow-wavelength light-emitting diodes (LEDs) [48–50,65,66].

### (a) Colour response of thrips

True colour vision is defined as the ability to discriminate colour (i.e. wavelength composition) independently from intensity [67,68]. For true colour vision, an animal must have at least two different photoreceptor classes, each with a different spectral sensitivity curve whose relative stimulations can be compared downstream with further neural processing by an opponent mechanism [1,69,70]. However, even if an animal displays different behavioural responses to different spectra, that does not necessarily mean it possesses true colour vision, but could instead be a display of wavelength-specific behaviours [67]. If no opponent mechanism is present, the innate and fixed action spectrum of such wavelength-specific behaviours is expected to match the spectral sensitivity of the responsible photoreceptor or photoreceptors [67,71,72].

Some studies suggest that thrips are highly attracted to certain ‘pure’ colours with narrow spectral curves. Strong attraction to yellow (570–590 nm), blue (420–470 nm) and UV (less than 400 nm) stimuli has been demonstrated in various thrips species (figure 5; electronic supplementary material, table S1). Yellow colour is thought to provide a ‘supernormal stimulus' to various species of phytophagous insects [1,73] due to strong, independent excitation to the green receptor, especially from bright yellow stimuli [74]. Blue and some parts of the violet region of the light spectrum (420–470 nm) have also been reported to strongly attract and catch high numbers of *F. occidentalis* and *Frankliniella tritici*, the eastern flower thrips [75–79]. Nonetheless, not all blues are equally attractive. For example, *F. occidentalis* prefers saturated hues of blue with narrower spectral curves that do not include significant wavelengths beyond 500 nm [75]. UV-A light (315–400 nm) is also attractive to thrips in the few instances that it has been tested independently [49]. Furthermore, the phytophagous pest *C. phaseoli* has even been shown to be sensitive to UV-B radiation (below 315 nm) in field and laboratory behavioural experiments [35,80].

The least favoured colours for most thrips include cyan and red, while the response to green tends to be more variable depending on the species and conditions in which the studies were conducted (electronic supplementary material, table S1; figure 5). Colours with a peak wavelength of approximately 490–530 nm are unattractive for *F. occidentalis* compared to blue [78]. Red is also usually poorly attractive for thrips species [79]. This reduced behavioural response to red is consistent with the low sensitivity observed from thrips retina at wavelengths above 600 nm [48–50]. Interestingly, species of thrips predominantly living and breeding on grasses, such as *Frankliniella tenuicornis* and some species of *Limothrips*, show little to no preference for any particular colour in field choice experiments [64,81–83]. It is not clear if the absence of colour preference in these species is the result of an equal preference for all tested colours or a lack of discrimination capabilities.

There is some evidence that blended colours with broad spectral curves or multiple peaks from different areas of the spectrum are either especially attractive or unattractive to thrips in certain contexts. For instance, using LEDs to mix green light (peak 523 nm) with a blue light (peak 467 nm), decreased the numbers of *F. occidentalis* caught compared to either blue or green alone [84]. On the other hand, blended yellow and UV stimuli are highly attractive to some thrips [77–79]. Similarly, various species of flower-dwelling thrips, such as *F. occidentalis*, *F. tritici*, *Thrips obscuratus* (New Zealand flower thrips) and species associated with foliage such as *T. tabaci*, exhibit a strong attraction towards non-UV-reflecting white (white −UV) compared to the unattractive UV-reflecting white (white +UV) [76–79,81,83,85,86]. If we assume that thrips are UV/blue/green trichromats, similar to honeybees, the mix of UV-A and yellow acts as a chromatic cue for thrips, equivalent to the ‘bee purple’ [79,87]. Conversely, UV-reflecting white is an achromatic cue for thrips because all three receptors are equally stimulated [87,88].

The variable responses of thrips to blended colours may be indicative of an opponent colour vision mechanism similar to those described for other insects [67,89–92], including herbivorous pests like aphids [10,27,93] and whitefly [54]. In aphids, for example, the high attractiveness of yellow is thought to be based on the high photon catch ratio between excitatory green photoreceptors and inhibitory blue receptors [10,94].

### (b) Intensity, contrast and background colour

Beyond wavelength composition, intensity is also of consequence to thrips response to light. Several studies in *F. occidentalis* demonstrate that increasing reflectance or a higher intensity of attractive colours like blue, yellow and white (−UV) increases attraction as determined by catch numbers, while increased intensity of other colours like green and white (+UV) do not change attraction levels [65,76–79,84] (electronic supplementary material, table S1). Red stimuli require intensities 100 times higher than yellow or blue to elicit positive phototaxis for *F. occidentalis* and *T. palmi* [48,66]. Furthermore, the response of thrips seems to reach a plateau at certain intensities, and the increase in attraction is not the same across the light spectrum, reinforcing the importance of colour composition [9,48,50,66].

The role that colour contrast and colour constancy play in attraction has not been widely studied in thrips. For *F. occidentalis*, stimuli that present high contrast with their background are typically more attractive. When yellow traps are used, black, violet or blue backgrounds improve trap effectiveness [81,95,96]. However, it is not clear if this is due to intensity or colour contrast effects. Indeed, there is currently no good evidence in thrips about the relative contribution of achromatic versus chromatic channels for their contrast sensitivity and visual response. It is possible that colour-intensity differences between blue and yellow are directly related to how attractive they appear to *F. occidentalis* in specific contexts.

Moreover, there is some evidence that coloured traps with complex three-dimensional structures, like ruffled cloth, may improve catch rates by creating contrasting areas of bright colour and dark shadows [97]. Certainly, there is much potential to improve our understanding of how thrips are attracted to patterns of contrasting colours and intensities. Also, how the ambient lighting environment impacts their attraction to these stimuli is not understood. For instance, in protected crops that are grown under greenhouse conditions or tunnels, some percentage of UV light can be filtered out of the environment by glass and other materials such as plastic films, causing disorientation in pest insects [98–100], a phenomenon that has so far been understudied.

### (c) Visual ecology of thrips

Many insects rely on visual cues in their search for suitable host plants for food and oviposition sites [1,17,73]. Locating the right host is important for thrips, as different host plants offer different nutritional value to adults and especially to the immature stages of their offspring that are otherwise unable to fly in search of alternative food sources. At longer distances, where thrips are limited by their poor visual resolution, olfaction is thought to be an important cue for thrips [11,97,101–103]. At shorter distances, colour and other visual cues become more important [104], but even then, the odour-colour multi-sensory interaction is still likely to be crucial for host-seeking behaviour [97].

Differences in host plant association may influence the colour attraction rates of different thrips species. For instance, grass-feeding thrips species like *Limothrips denticornis* and *Stenothrips graminum* demonstrate some degree of attraction to green colour [81]. Conversely, other studies evaluating coloured traps show that various species of flower-inhabiting thrips and non-grass-feeding thrips display little attraction to green, especially when other choices such as yellow, blue or non-UV-reflecting white are available (figure 5; electronic supplementary material, table S1). Nonetheless, phytophagous species of thrips oviposit and feed on green foliage, so it would be expected that green colour is still important for thrips, at least to localize plants in general. In addition, it is possible that a yellow stimulus can cause a high stimulation of the green receptor in insects, if it has a substantially higher intensity than a green stimulus [74]. Less commonly but in the same manner, red is typically one of the least attractive colours for thrips, although *Thrips simplex* shows a preference for orange-red [79], and *Frankliniella schultzei* shows a specific attraction towards red, which is similar to the flower colour of its preferred host [105,106].

Colour preference in thrips can also be affected by season [107]. For example, *Thrips angusticeps* shows strong attraction towards yellow colour before flowers bloom and then changes preference towards blue and white when flowers are in full bloom [81]. In addition, there is some evidence suggesting that the response of thrips to colour could also be correlated with light fluctuations between different times of year [108]. However, it is not clear whether natural light fluctuations between seasons have a direct effect on *F. occidentalis* behaviour in that case, or if the change in response is related to changes in light reflected from surfaces. Lastly, even though some differences in colour attraction between female and male thrips have been found [106], normally colour attraction for both sexes tends to be similar [76–78,82,83,85].

Many published studies clearly illustrate that thrips have colour discrimination capabilities and, in some species, colour preferences can be associated with their ecology. What remains to be determined is how good thrips colour discrimination is and whether they indeed possess true colour vision. The degree of attraction to a colour varies among different thrips species, although intra-specific variation of colour attraction is also found in widely studied species like *F. occidentalis* [109]. Unfortunately, many of the behavioural studies evaluating thrips colour attraction are based on spontaneous behaviours including orientation and landing on different coloured stimuli, which limits interpretation of results in a broader context. This emphasis is probably because studies have been conducted with a pest management objective, putting little attention into understanding the main drivers of thrips response to colour or to the relationship between thrips colour vision, their ecology and their natural history. In addition, drawing sound conclusions from such behavioural studies on colour attraction can be problematic because not all studies provide spectral data of the colour stimuli used in the experiments and colours evaluated across different studies vary in spectral quality (i.e. differences in hue, percentage of spectral reflectance and shape of reflectance curves, intensity measured in different ways, etc), in many cases confounding wavelength composition and intensity.

### (d) Colour vision and pest management of thrips

From an insect pest management perspective, identifying the optimal colour to use as an attractant is fundamental for monitoring purposes as an early warning when there are very low numbers present, or for pest control measures for very large populations. However, for *F. occidentalis*, arguably the most-studied thrips species, there is still no consensus on whether the traditional blue or yellow stimuli, or some other blend of colours, are the best to significantly increase thrips captures for control practices such as mass trapping, lure and infect or lure and kill [97,110]. In the majority of cases, yellow sticky traps have a light reflectance peak of approximately 80%, while most of the blue traps do not reach more than 50%, with some exceptions reaching peak reflectance higher than 60% and highly attractive blues around 70% [77]. When using LEDs under varying intensities in dark conditions with no ambient light in a laboratory set-up, blue (470 nm) was found to attract significantly higher numbers of *F. occidentalis* than yellow (590 nm) at the same 40 lux of intensity [9]. When both blue and yellow LEDs were evaluated to higher intensities of 80 and 100 lux, yellow became more attractive and caught significantly higher numbers of *F. occidentalis* than blue [9]. More work is needed to disentangle the ideal chromatic composition, intensity and spatial patterns for attracting thrips. Furthermore, the placement of traps in the environment needs to be considered in greater detail in order to present stimuli at ideal orientations and with ideal backgrounds to maximize their attractiveness. Finally, additional scrutiny needs to be placed on experimentation in relevant lighting conditions. Efforts should be made to create controlled laboratory set-ups that better mimic the light environments of the greenhouses or fields where traps are intended to be used.

Many older studies that used reflecting surfaces such as coloured sticky traps as visual stimuli confounded wavelength composition and percentage of light reflected, making it hard to determine if attraction or lack thereof was a result of just colour, or if light intensity had also contributed. During the last decade, studies evaluating phototactic behaviour in thrips have improved spectral accuracy by using narrow-band, intensity-adjustable LEDs as the visual stimulus [3,9,35,48,50,65,66,84]. Compared to the broader spectral curves and poorly controlled intensities of coloured surfaces that rely on the interaction with ambient light, LEDs emit narrower wavelength ranges and can be adjusted for intensity. Nonetheless, studies using LEDs tend to corroborate previous findings from older studies, showing that blue and yellow are among the most attractive colours to flower- and foliage-feeding thrips, including important pests such as *F. occidentalis* and *T. tabaci* [9,49,65,66,84] (figure 5; electronic supplementary material, table S1). Nevertheless, intra-specific variation in colour attraction is still observed in the economically important *F. occidentalis* across studies, with a clear dichotomy between blue and yellow (figure 5).

For deployment of coloured lures in a pest management context, additional considerations related to the mechanism of trapping must also be addressed. For instance, Van Tol *et al*. [108] found that the degree of haziness of different glues used for sticky traps can change the blue and yellow preference in *F. occidentalis*. Specifically, a hazy glue makes blue traps more attractive than yellow ones, whereas a more transparent glue makes yellow traps more attractive than blue ones. It is possible that the glues cause subtle changes in wavelength composition of the reflective surfaces that could be detected if *F. occidentalis* does indeed possess fine opponent colour vision. However, it must also be considered whether the different glues produce confounding polarization patterns or contain odorants that are discernible by thrips.

## 4. Conclusion

Thysanoptera is an ecologically diverse group of insects with remarkable eye adaptations despite their small size. The study of thrips vision has mainly focused on pest species of thrips and their attraction to coloured traps. However, there are still large knowledge gaps regarding the optical structure and function of thrips' compound eyes. In order to properly test colour stimuli, it is crucial to gather more information about thrips eye physiology, specifically the precise spectral sensitivity curves of their individual photoreceptors via intracellular recordings. With this information, models describing thrips colour vision can then be developed and tested to help predict how best to use or manipulate colour, contrast and intensity for pest management purposes. Furthermore, studies to explore opsin expression as well as the function and ecological relevance of the diverse putative spectral filters observed in thrips ventral ommatidia, and investigations of additional thrips species, are needed to better understand the contribution of localized retinal specializations towards the visual ecology of these insects. In addition, there is a rich amber record of fossilized species of Thysanoptera [111–115], raising the tantalizing possibility of discovering the evolutionary history of compound eye adaptations in this intriguing insect group.

A large range of experiments over the last half-century have shown that various species of thrips are able to discriminate different colours. However, whether thrips possess true colour vision or if their colour attraction is a result of wavelength-specific behaviours is still unclear [67]. Furthermore, colour attraction needs to be better considered in light of other attractive visual effects such as intensity, pattern, background and colour constancy. The trial-and-error approach used in many studies, coupled with the lack of proper controls for light intensities and deficiencies in carefully reporting spectral properties of stimuli has led to inconsistent results among studies. Complicating things further, differences in methodology, lighting context in which the insects were tested, time of the year, host plant effects and intra-specific variation are rarely considered. All these shortcomings have proven to be problematic towards understanding colour vision in thrips and make it difficult to draw sound conclusions from the behavioural studies that evaluate their colour attraction.

A more systematic approach is needed to understand the fundamentals of thrips colour vision so that we can identify the optimal stimuli for attracting pest species for monitoring and mass trapping [97,109]. This has improved in recent years, but standardization of methods is still needed to resolve ambiguities. The availability of LEDs has allowed researchers to control for factors like light intensity and evaluate the effect of specific wavelengths of light on thrips behaviour. However, many of these experiments are performed in dark or otherwise unnatural experimental set-ups. Ultimately, these approaches need to be adapted to simulate relevant lighting environments and be assessed in field trials. Also, additional attention should be paid to the visual behaviour of thrips prior to landing and getting stuck in a glue trap. Is vision used to detect foliage at a distance, and at what point do they decide to land? This can influence optimal lure placement and orientation. Finally, other important senses—such as olfaction—also need to be considered. Visual and olfactory cues together have been shown to have an important interaction in thrips behaviour [97,102,116]. Thus, a more ecologically oriented approach might prove to be useful to address questions about what thrips are looking for, how they might be using their vision and other senses in concert to find it, and the specific ecological contexts that drive their behaviours.

With new technologies such as micro-computed tomography, serial block-face electron microscopy and environmental light field measurement standards [117] becoming increasingly available, it will be easier to tackle the challenge of working with such small animals. Three-dimensional modelling of thrips vision in simulated environments facilitated by these approaches will help us to answer questions about their visual perception and unravel the mechanisms behind their colour vision. Further, real-time three-dimensional tracking and high-speed videography could allow us to examine decision making in free-flying thrips as they approach visual stimuli. New advances along these lines will allow us to better inform the development of trapping systems that aim to use colour-related cues to significantly increase detection and attraction of thrips and maybe even other pest insects.

## Data Availability

All relevant data is included in the main text or provided in the electronic supplementary material [118].
